# Genetic and environmental pathways to complex diseases

**DOI:** 10.1186/1752-0509-3-46

**Published:** 2009-05-05

**Authors:** Julia M Gohlke, Reuben Thomas, Yonqing Zhang, Michael C Rosenstein, Allan P Davis, Cynthia Murphy, Kevin G Becker, Carolyn J Mattingly, Christopher J Portier

**Affiliations:** 1 Environmental Systems Biology Group, Laboratory of Molecular Toxicology, National Institute of Environmental Health Sciences, Research Triangle Park, NC 27709, USA; 2 Gene Expression and Genomics Unit, National Institute on Aging, National Institutes of Health, Baltimore, MD 21224, USA; 3 Department of Bioinformatics, Mount Desert Island Biological Laboratory, Old Bar Harbor Road, Salisbury Cove, ME 04672, USA

## Abstract

**Background:**

Pathogenesis of complex diseases involves the integration of genetic and environmental factors over time, making it particularly difficult to tease apart relationships between phenotype, genotype, and environmental factors using traditional experimental approaches.

**Results:**

Using gene-centered databases, we have developed a network of complex diseases and environmental factors through the identification of key molecular pathways associated with both genetic and environmental contributions. Comparison with known chemical disease relationships and analysis of transcriptional regulation from gene expression datasets for several environmental factors and phenotypes clustered in a metabolic syndrome and neuropsychiatric subnetwork supports our network hypotheses. This analysis identifies natural and synthetic retinoids, antipsychotic medications, Omega 3 fatty acids, and pyrethroid pesticides as potential environmental modulators of metabolic syndrome phenotypes through PPAR and adipocytokine signaling and organophosphate pesticides as potential environmental modulators of neuropsychiatric phenotypes.

**Conclusion:**

Identification of key regulatory pathways that integrate genetic and environmental modulators define disease associated targets that will allow for efficient screening of large numbers of environmental factors, screening that could set priorities for further research and guide public health decisions.

## Background

Determining the extent to which environmental versus genetic factors are responsible for particular phenotypes is a central question in all of biological research. Elucidating associations between genotype and phenotype has been a central goal in human health research for some time, and has resulted in an impressive collection of research on genotype-phenotype relationships [1,2]. While continued analysis of rare monogenic phenotypes is important for mechanistic discoveries [3], unraveling the interplay between genetic and environmental determinants of complex phenotypes will be critical for improving public health [4]. For example, gene-environment interactions have been shown to play a critical role in childhood leukemia and asthma [5-7]. However, much less is known about gene-environment interactions as they relate to the etiology of the common complex disease phenotypes such as unipolar depressive disorders, ischemic heart disease and cerebrovascular disease, all of which fall within the top six causes of the global burden of disease, and are projected to increase as the epidemiological transition continues in developing countries [8].

Network and bioinformatic methods have recently been applied to synthesize data on gene-disease relationships for those diseases that have a strong genetic component [9-11]. In addition, utilization of functional information to prioritize candidate driver genes in cancer has been advocated [12]. However, application of network theory to determine the interplay between genetics and environmental factors in complex diseases has been left unexplored. We hypothesize genetic and environmental factors involved in the progression of a particular complex phenotype are participants in the same underlying cellular processes. To test this hypothesis, we develop networks of complex diseases and environmental factors through linkage of human genetic association studies and mechanistic analyses of environmental factors, using evolutionarily conserved molecular pathways as the unifying system to define relationships. We further explore relationships identified by this method through comparison to known disease-chemical relationships and analysis of transcriptional regulation in gene expression datasets for metabolic syndrome phenotypes, neuropsychiatric phenotypes and several predicted environmental modulators.

## Results and discussion

### Clustering phenotypes by pathways

**Figure 1:**
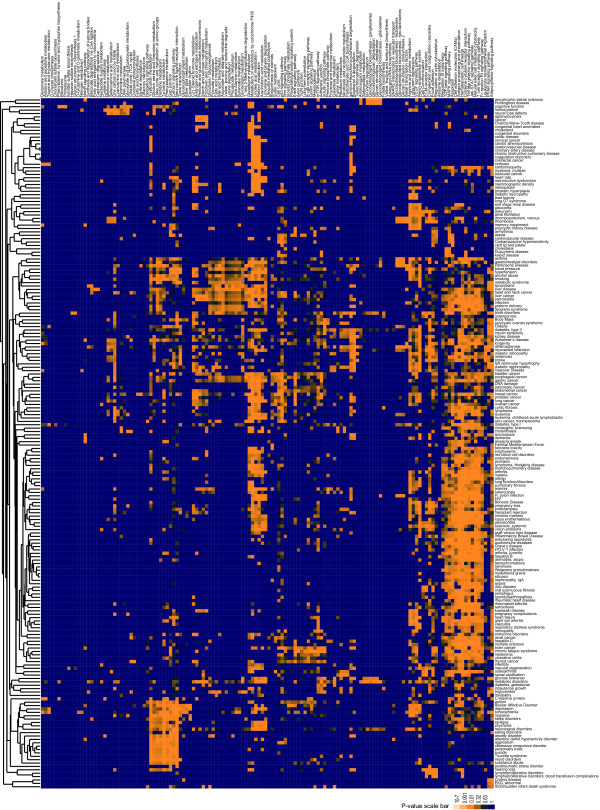
**Unsupervised Hierarchical Cluster of Phenotypes by Pathways**. Genes associated with a particular phenotype were evaluated for enrichment in KEGG pathways using SEPEA. P-values for KEGG pathway enrichment were then clustered using Spearman rank correlation in Cluster and the graphic was prepared using TreeView [73], where color ranges linearly from blue (p = 1) to orange (p = 0). Phenotype-gene relationships were downloaded from the Genetic Association Database [1] in June 2007 and phenotypes were further grouped according to Additional file 1. Request the TreeView file of this cluster from Julia Gohlke gohlkej@niehs.nih.gov for more detailed exploration.

To identify common pathways between complex diseases, we annotated gene-phenotype relationships found in the Genetic Association Database (GAD) [1] (see Additional File 1), then analyzed these phenotype-associated gene lists using the Structurally Enhanced Pathway Enrichment Analysis (SEPEA) algorithm (See methods for summary and described in detail in [13]). This resulted in a clustergram of disease phenotypes based on the underlying pathways that are represented by the sum of polymorphic genes associated with a particular phenotype (See Methods) (Figure 1). Distinct clusters of phenotypes with similar broad clinical characteristics are evident such as cancers, cardiovascular and metabolic diseases, immune-related disorders, and neuropyschiatric disorders. Furthermore, the pathways that define these clusters are consistent with our current understanding of disease etiology. For example, the cancer cluster is defined by low p-values for Erbb, p53, and cell cycle pathways and the neuropsychiatric cluster is defined by low p-values for neuroactive ligand receptor interactions, calcium signaling, as well as tryptophan and tyrosine metabolism. Moreover, immune related pathways (e.g. Jak-STAT signaling, Toll-like receptor signaling, T cell receptor signaling etc.) contribute not only to classic autoimmune and infectious disease phenotypes, but also to a large proportion of the divergent phenotypes represented, such as cardiovascular and cerebrovascular disorders, kidney disease, as well as Alzheimer's disease and longevity, among others. Several unexpected results are also evident, such as the co-clustering of pregnancy loss and preeclampsia with immune phenotypes such as lupus erythematosus and Behcet's Disease and the co-clustering of asthma with Parkinson's disease.

Interesting relationships are observed through a comparison of pathways that are associated with preclinical phenotypes to those pathways that are significantly associated with outright disease. For example, when we look at common neuropsychiatric disorders, such as depression and anxiety disorder, we see that genes associated with these phenotypes are specifically associated with neuroactive ligand receptor interactions, calcium signaling, as well as tryptophan and tyrosine metabolism. However, we see that these pathways significantly associated with neuropsychiatric disorders are also associated with obesity, hypertension, and blood lipoprotein composition as well as substance abuse and smoking, all of which are significant risk factors for heart disease [14]. In contrast, genes associated with outright disease phenotypes (e.g. vascular disease, heart failure, myocardial infarction, and stroke), are significantly enriched in cardiovascular specific pathways such as the renin-angiotensin system and the VEGF signaling pathway, as well as immune related pathways, suggesting genetic susceptibility to outright heart failure can be distinguished from genetic susceptibility to risk factors for development of heart disease. Therefore, this phenotype-pathway cluster of genetic associations can delineate pathways that may be important at different points in the progression of complex chronic diseases.

### An interaction network of phenotypes and environmental factors

Next, we sought to meld current knowledge of genetic susceptibility factors with environmental factors that contribute to a particular complex phenotype. To accomplish this, we identified enriched pathways based on compiled lists of environmental factor-gene/protein relationships described in the Comparative Toxicogenomics Database [15]. Networks between phenotypes (using genetic association studies as described above) and environmental factors were then developed where edges represent at least 2 significant pathways between a given phenotype-phenotype, phenotype-environmental factor, or environmental factor-environmental factor pair. In addition, 8 categories including neoplastic, cardiovascular, metabolic, immune, endocrine, neuropsychiatric, pulmonary, and hematologic were used to more broadly define phenotypes. These broad categorizations are important as many of the relationships found in the Comparative Toxicogenomics Database are derived from animal models.

**Figure 2:**
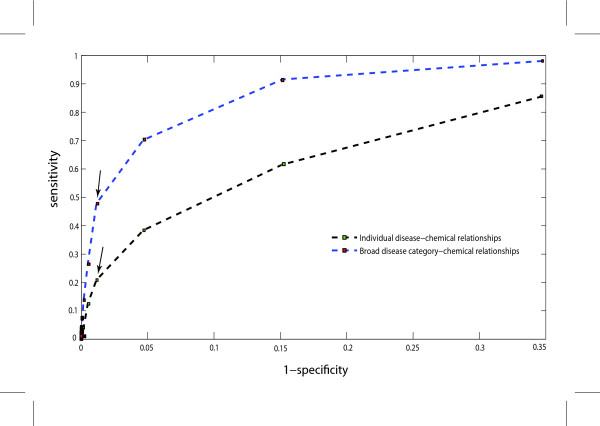
**Receiver operating characteristic (ROC) curve**. A graphical representation of the sensitivity versus (1-specificity) comparing environmental factor-phenotype predictions at different p-value cutoffs to a manually curated set of direct chemical-disease relationships from the Comparative Toxicogenomics Database [16] using either specific diseases or broad categorizations of diseases. The SEPEA pathway enrichment p-value cutoff of 0.003 is indicated with arrows for each analysis.

We compared our predicted phenotype-environmental factor relationships to a set of 1084 manually curated direct chemical-disease relationships as reported in the Comparative Toxicogenomics Database [16]. The receiver-operator curve (ROC) is illustrated in Figure 2. This figure suggests the relative loss in specificity outweighs the gain in sensitivity at a SEPEA pathway enrichment p-value cutoff of approximately 0.003 for both specific and broad categorizations of environmental factor-phenotype relationships. At this p-value cutoff, 226 of the 10,793 predicted environmental factor-phenotype relationships are supported by manually curated evidence, demonstrating the majority of connections within our network define new hypotheses of environmental factor-phenotype relationships, yet this overlap is much higher than would be expected by chance (p < 10^-16^). When the diseases analyzed are collapsed into the eight broad disease categories, 48% (or 271 out of 567) of the manually curated relationships are captured in our analysis (p < 10^-16^). This suggests that our method is more sensitive in identifying known chemical-disease category relationships than in identifying known specific disease-chemical relationships. This result makes sense in light of the fact that environmental factor data is largely derived from animal models, where one would not predict strong concordance between phenotypes and specific human diseases. In addition, the pathways analyzed are not specific to tissue and/or life stage, suggesting a specific disease of a particular tissue or developmental stage will be hard to differentiate using this method. Based on the hypothesis that there are common pathways associated with both the genetic and environmental components of broad disease categories.

**Figure 3:**
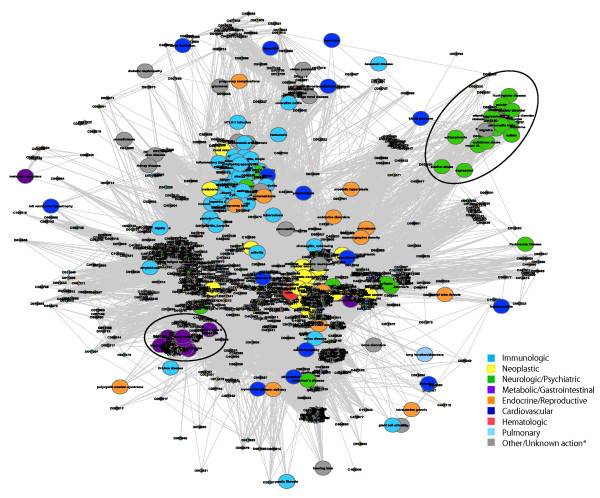
**Interaction Network of Phenotypes and Environmental Factors**. Phenotypes are represented as circular nodes and environmental factors as diamond shaped nodes. Edges represent sharing at least two significantly enriched pathways (p ≤ 0.003) using lists of genes associated with a particular phenotype or environmental factor, according to the phenotype-gene relationships in the Genetic Association Database [1] or the environmental factor-gene relationships found in the Comparative Toxicogenomics Database[15], respectively. MeSH IDs are used as environmental factor node labels. Environmental factors with pharmacological or toxicological action in the MeSH record are color coded based on broad phenotype categories according to annotation in Additional file 2. *Phenotypes which do not fit into a specific category or environmental factors with undetermined pharmacological or toxicological action are gray. Request the Cytoscape session file of this network from Julia Gohlke gohlkej@niehs.nih.gov for more detailed exploration. Annotation of the circled metabolic syndrome and neuropsychiatric subnetworks can be found in Additional file 3.

A graphical representation of the predicted network is presented in Figure 3, where environmental factors with known physiological actions are colored coded based on MeSH annotation (see Additional File 2 for complete annotation of nodes). Because the above comparison determined the sensitivity and specificity of our environmental factor-phenotype predictions based on only those 1084 chemical-disease relationships manually curated within CTD, we wanted to determine if the phenotype-phenotype, environmental factor-environmental factor, and environmental factor-phenotype relationships predicted in this graph are supported by known broad categorizations of phenotypes and physiological actions of the environmental factors. Therefore, we computed the significance of the number of edges that are shared between nodes in a given category using the graph clustering coefficient [17]. Using this method, the clustering of the metabolic, immune, neoplastic, and neuropsychiatric phenotypes are considered significant (p ≤ 0.05). However, when the MeSH annotated environmental factors are added, only the immune and neoplastic categories are significant (p ≤ 0.05), suggesting the broad categorization used may not be suitable to describing endocrine and cardiovascular phenotypes, or the MeSH annotated physiological actions of many of the environmental factors in this network.

An important application of this work is generating hypotheses of interacting environmental factors that may be important in the prevention, initiation, progression, or treatment of complex diseases based on the network relationships found between phenotypes and environmental factors. Therefore, the tight cluster of metabolic syndrome phenotypes and neuropsychiatric disorders identified in Figure 3 are examined in further detail through analysis of gene expression datasets.

### Metabolic syndrome cluster

Significance in both PPAR signaling and adipocytokine signaling form the tight subnetwork of 93 environmental factors linked to several metabolic syndrome phenotypes such as serum lipoprotein and triglyceride levels, body mass index, insulin sensitivity, type II diabetes, and obesity (see Additional File 3). Consistent with our results, a recent network analysis of microarray datasets from diabetes patients suggests PPAR signaling is the key underlying pathway in the pathogenesis of Type II diabetes [18]. Thiazolidinediones, which are antidiabetic PPARγ agonists [19,20], the PPARα agonist fenofibrate and the HMG-CoA reductase inhibitor atorvastatin, both of which are used in the treatment of hyperlipidemia [21,22] are identified in this subnetwork. Furthermore, dopamine antagonists, which includes several antipsychotic medications known to cause weight gain [23] are identified in this cluster. Retinoids are also found in this cluster, which is particularly intriguing in light of novel research showing retinaldehyde represses diet-induced obesity [24]. In fact, the widely used antineoplastic synthetic retinoic acid receptor alpha agonist Ro 41–5253 has recently been shown to induce PPARγ activity [25]. In addition, the increasing body of evidence linking Body Mass Index, retinoids, and cancer risk was recently highlighted in the most comprehensive analysis to date on diet and cancer risk [26]. In addition to the pharmaceuticals identified, di-n-hexyl phthalate (DHP), a widely used plasticizer that has recently been shown to act as a PPAR agonist [27] in addition to previous findings that high levels of exposure cause reproductive toxicity in animal models [28]. The Omega 3 fatty acids present in fish oil are an important dietary environmental factor identified in this cluster [29].

**Figure 4:**
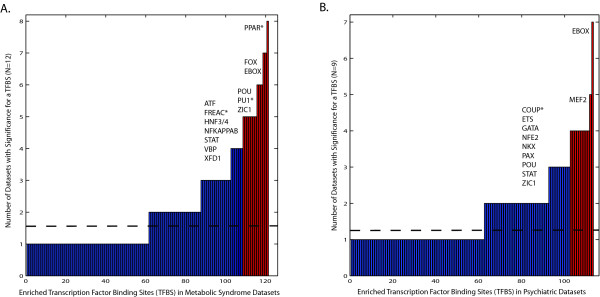
**Gene expression regulation across microarray datasets**. Enriched transcription factor binding sites (tfbs) were identified in evolutionarily conserved regions surrounding differentially up and downregulated genes from metabolic (A.) or neuropsychiatric (B.) microarray datasets identified in Table 1 (see Methods). Results for each microarray dataset are presented in Additional file 4. The mean frequency of identifying a particular tfbs enriched in a dataset was 13% (dotted line). Those enriched tfbs that are consistently identified across the metabolic (A.) or neuropsychiatric (B.) datasets are color coded red (p ≤ 0.005), whereas those tfbs that are specific to the metabolic datasets versus neuropsychiatric datasets and vice versa (p ≤ 0.05) are identified with an asterisk.

To test the hypothesis that regulation via PPAR and adipocytokine signaling plays an important role in environmental and genetic factors influencing metabolic syndrome phenotypes, we analyzed gene expression datasets after exposure to several predicted environmental modulators, as well as gene expression datasets from Familial combined hyperlipidemia cases, obese versus lean Pima Indians and obese versus lean mice fed a controlled diet (Table 1) [30-44]. Lists of significantly up or down regulated genes were submitted to DiRE , a transcription factor binding site (tfbs) enrichment optimization algorithm that identifies tfbs that are enriched in evolutionary conserved regions surrounding a given set of genes versus a randomly generated background set of genes [45]. Lists of the tfbs enriched in the evolutionarily conserved regions surrounding the significantly up or downregulated gene list for each dataset are compiled in Additional file 4. Across all of these independent datasets, binding sites for the three transcriptional regulators of PPAR and adipocytokine signaling, namely PPAR, NFkB, and STAT, are consistently enriched in the differentially expressed gene sets (p ≤ 0.005) (Figure 4A). Therefore, this alternative analysis supports our previous subnetwork predictions suggesting a variety of environmental factors as well as genetic contributions to metabolic syndrome phenotypes can be integrated at the level of PPAR and adipocytokine signaling pathways. When the enriched tfbs identified for these metabolic syndrome subnetwork datasets are compared to enriched tfbs identified in the neuropsychiatric datasets (described below), we see that PPAR, PU.1 and FREAC binding sites are significantly enriched in these metabolic syndrome datasets (p ≤ 0.05).

**Table 1:** Global gene expression datasets utilized for validation of metabolic syndrome and neuropsychiatric subnetworks

**METABOLIC SYNDROME**				
***Condition***	***Species***	***Tissue***	***GEO Acc***.	***Reference***

obese/lean	Human	adipocytes	GSE2508	[30]
obese/lean	Mouse	adipocytes	GSE4692	[31]
Familial combined hyperlipedemia	Human	monocytes	GSE11393	[32]
				
***Treatment ***	***Species ***	***Tissue ***	***GEO Acc***.	***Reference ***

Fenofibrate	Rat	liver	GSE8251	[33]
4-hydroxyphenylretinamide	Rat	liver	GSE3952	[34]
9-cis retinoic acid	Rat	liver	GSE3952	[34]
Targretin	Rat	liver	GSE3952	[34]
Vitamin A deficient diet	Rat	liver	GSE1600	[35]
Omega 3 fatty acids	Rat	cardiomyocytes	GSE4327	[36]
Thiazolidinediones	Human	3T3-L1 adipocytes	GSE1458	[37]
Atorvastatin	Human	monocytes	GSE11393	[32]
Cyfluthrin	Human	astrocytes	GSE5023	[38]
				
**NEUROPSYCHIATRIC DISORDERS**				

***Condition ***	***Species ***	***Tissue ***	***GEO/EBI Acc***.	***Reference ***

Bipolar Disorder	Human	prefrontal cortex	GSE12654	[39]
Depression	Human	prefrontal cortex	GSE12654	[39]
Schizophrenia	Human	prefrontal cortex	GSE12654	[39]
Schizophrenia	Human	frontal cortex	E-MEXP-857	[40]
Anxiety	Mouse	various brain regions	GSE3327	[41]
Autism	Human	lymphoblastoid cell lines	GSE7329	[42]
Autism	Human	whole blood	GSE6575	[43]
				
***Treatment ***	***Species ***	***Tissue ***	***GEO Acc***.	***Reference ***

Chlorpyrifos	Human	astrocytes	GSE5023	[38]
Chlorpyrifos	Rat	forebrain	GSE9751	[44]

Other tfbs beyond PPAR and adipocytokine signaling regulators that are highly enriched across these datasets offer hypotheses for future experimental research in the transcriptional regulation of metabolic syndrome phenotypes. For example, EBOX sites for basic helix-loop-helix transcription factor and PU.1, an ETS like tf, are important in cell fate programs in hematopoesis, particularly in the monocyte/macrophage lineage [46,47]. This is intriguing in light of numerous studies showcasing the importance of macrophages not only in cardiovascular disease, but in the development of obesity as well[48,49] and their connectivity to PPAR signaling [50,51]. ZIC1 is a zinc finger transcription factor known to be important during early developmental programs [52], while preliminary genetic association work suggests ATF/CREB tfs may also play a role in obesity[53]. Finally, FREAC sites bind several forkhead members (FOXF2, FOXC1, FOXD1, AND FOXL1), which have been shown to be important in the regulation of gut-associated lymphoid organ development and regulation of intestinal glucose uptake in mice [54,55].

### Neuropsychiatric cluster

Our results suggest data from genetic association studies for several neuropsychiatric diseases (autism, schizophrenia, depression, bipolar disorder, attention deficit hyperactivity disorder, anxiety disorder, obsessive compulsive disorder, and Huntington's disease) converge on tyrosine metabolism and neuroactive ligand receptor interactions, forming a tight cluster of these phenotypes linked by significance in these two pathways. In fact, genes that code receptors and metabolic enzymes of the dopamine and serotonin signaling systems form the basis of this result. In contrast to the metabolic syndrome cluster, very few environmental factors (11) are found in this tight cluster and include the opiate pentazocine, the muscarinic receptor agonist pilocarpine, and the GABA modulator pentobarbital (Additional File 3). In addition, the acetylcholinesterase inhibiting organophosphates, well known for their use as pesticides, are identified in this cluster.

We analyzed gene expression datasets from case versus control studies for several of the phenotypes, as well as gene expression datasets generated from fetal astrocytes or rat forebrain after exposure to the organophosphate pesticide chlorpyrifos (Table 1). Following the method described for analysis of the gene expression datasets for the metabolic syndrome cluster, lists of significantly up or down regulated genes were submitted to DiRE [45]. Lists of enriched tfbs in regions surrounding the significantly up or downregulated gene lists for each dataset are available in Additional file 4. Across all of these datasets, enrichment for EBOX and MEF2 binding sites are found most consistently in the differentially expressed genes for the neuropsychiatric cluster datasets (Figure 4B). Consistent with the result, several studies suggest coordinated action of the EBOX binding proneural bHLH transcriptional activators and Mef2c in the differentiation of neuronal subtypes in the developing mammalian forebrain [56-59]. When the enriched tfbs identified for these neuropsychiatric subnetwork datasets are compared to enriched tfbs identified in the metabolic syndrome datasets described above, we see that only chicken ovalbumin upstream promoter transcription factor (COUP) binding sites are significantly enriched in neuropsychiatric datasets (p ≤ 0.05). COUP-TFs are members of the steroid receptor superfamily in which dopamine is thought to be a physiological activator [60].

### Consideration of bias associated with genetic association studies

One potential source of bias is the likelihood of false positive associations represented in the GAD database. For example, a large multi-center study could not validate several previously reported genetic risk factors for acute coronary syndrome [61]. In addition, publication of false positive events could lead to more extensive publication bias as these results are followed up for related phenotypes. To address this potential for bias, we have re-evaluated 3 phenotypes for which extensive meta-analyses of genome-wide association (GWA) findings exist [62-64]. The list of genes from these recent meta-analyses of GWA studies for Alzheimer's Disease, Parkinson's Disease, and Schizophrenia represent a subset of those KEGG represented genes found within GAD, as only one novel gene for each phenotype was identified by the GWA meta-analysis across these phenotypes (Additional file 5). Subsequently, the SEPEA algorithm was re-run using the genes associated with each phenotype based on these meta-analyses of GWA results. In general, the predicted enriched pathways were consistent across results obtained with the GAD and GWA generated lists, however there were some notable differences (Additional file 5). For example, for both the GAD and GWA Alzheimer's Disease gene lists, the Renin-Angiotensin System pathway ranked the highest, however, using the results from the meta-analysis of GWA studies suggests folate related pathways may be important whereas the GAD results suggest tyrosine metabolism is altered. In fact, tyrosine metabolism ranks high for all three phenotypes using the GAD generated gene list, whereas Parkinson's Disease is the only phenotype in which the GWA studies confirm this result. As more GWA results become available for other phenotypes, this potential limitation of the current analysis can be more fully evaluated.

### Pathways to disease

**Figure 5:**
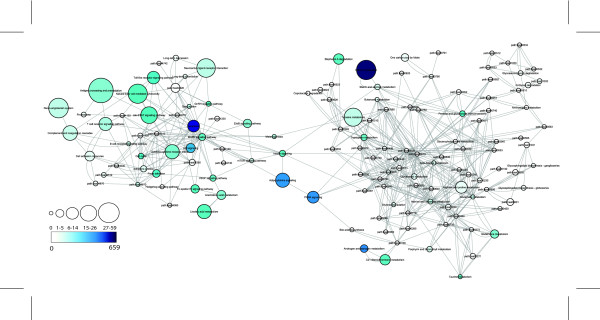
**Pathway Interaction Network**. Nodes represent KEGG metabolic and signaling pathways and are connected based on the KEGG database [72]. Node size is reflective of the number of phenotypes associated with the particular pathway based on application of SEPEA (p ≤ 0.003) to gene lists annotated from The Genetic Association Database[1]. Intensity of node color is reflective of the number of environmental factors associated with the particular pathway based on enrichment of gene lists annotated from The Comparative Toxicogenomics Database[15].

Ultimately, a particular phenotype is produced by the integration of outputs from a multitude of molecular pathways within an organism. Therefore, we explored the higher order structure of pathway networks by overlaying our analysis onto the network structure of interconnected KEGG pathways (Figure 5). This analysis allows us to simultaneously visualize the key pathways to complex disease progression from the genetic standpoint by adjusting node size to reflect the number of human phenotypes associated with a particular pathway based on the sum of disease associated genetic polymorphisms, as well as from the environmental standpoint, by adjusting the color of the pathway node to reflect the number of environmental factors associated with a particular pathway.

Looking at the intersection of the top 15 pathways most often enriched in genetic association studies and environmental factor research (Table 2), suggests metabolism of xenobiotics by cytochrome P450, retinol metabolism, Jak-STAT signaling, Toll-like receptor signaling, and adipocytokine signaling may be five critical pathways important to disease progression from both a genetic and environmental standpoint. From our analysis of phenotypes illustrated in Figure 1, we see that metabolism of xenobiotics by cytochrome P450 is significantly enriched in genetic association datasets for several phenotypes including cancers, cardiovascular disease, and immune related disorders. Adipocytokine signaling defines the cardiovascular and metabolic syndrome phenotypes, many of which have reached epidemic levels over the last 30 years [65], suggesting environmental components are critical in the etiology of these phenotypes. Retinol metabolism is significantly enriched in genetic polymorphism lists for hormonally regulated cancers such as breast, endometrial, testicular, prostate and thyroid, as well as pregnancy complications, reproductive dysfunction, and cardiovascular and endocrine disorders. This group of phenotypes is particularly interesting in light of the latest time trend statistics from the National Cancer Institute and Centers for Disease Control. As a whole, cancer incidence rates have been declining over the last decade, with the exception of 5 sites (thyroid, liver, kidney, skin, and testis). Thyroid cancer has by far the largest increase in incidence over the last decade, with an annual percent change of 5.3 between 1994 to 2004 [66]. In addition, pregnancy complications and endocrine disorders account for 5 of the 6 primary diagnoses with the greatest percent increase in ambulatory care visits over the last decade [67]. These time trends suggest environmental components are critical in rising incidence of endocrine related phenotypes, as the timeframe hardly crosses a generation, highlighting the importance of continued research on exposure routes and health effects of potential endocrine disrupters found in our environment [68,69].

**Table 2:** Top pathways enriched using genetic association research or environmental factor research.

**Top 15 pathways enriched using phenotype-gene research compiled in the Genetic Association Database**			
*Pathway Name *	*KEGG pathway ID *	*Number of diseases significantly enriched for pathway *	*Number of environmental factors significantly enriched for pathway *
Antigen processing and presentation	path:hsa04612	59	57
Metabolism of xenobiotics by cytochrome P450	path:hsa00980	49	421
Hematopoietic cell lineage	path:hsa04640	40	52
Renin-angiotensin system	path:hsa04614	28	29
Retinol metabolism	path:hsa00830	27	659
Natural killer cell mediated cytotoxicity	path:hsa04650	26	97
Neuroactive ligand-receptor interaction	path:hsa04080	24	30
Tyrosine metabolism	path:hsa00350	23	28
Jak-STAT signaling pathway	path:hsa04630	20	104
Complement and coagulation cascades	path:hsa04610	16	24
Linoleic acid metabolism	path:hsa00591	15	97
Cytokine-cytokine receptor interaction	path:hsa04060	15	63
Adipocytokine signaling pathway	path:hsa04920	15	185
T cell receptor signaling pathway	path:hsa04660	13	38
Toll-like receptor signaling pathway	path:hsa04620	12	114
			
**Top 15 pathways enriched using environmental factor-gene research compiled in the Comparative Toxicogenomics Database**			
			
*Pathway Name *	*KEGG pathway ID *	*Number of diseases significantly enriched for pathway *	*Number of environmental factors significantly enriched for pathway *

Retinol metabolism	path:hsa00830	27	659
Apoptosis	path:hsa04210	11	457
Metabolism of xenobiotics by cytochrome P450	path:hsa00980	49	421
gamma-Hexachlorocyclohexane degradation	path:hsa00361	7	224
Androgen and estrogen metabolism	path:hsa00150	5	194
PPAR signaling pathway	path:hsa03320	11	189
Adipocytokine signaling pathway	path:hsa04920	15	185
p53 signaling pathway	path:hsa04115	7	160
Toll-like receptor signaling pathway	path:hsa04620	12	114
Focal adhesion	path:hsa04510	5	114
Cell cycle	path:hsa04110	2	113
Pentose and glucuronate interconversions	path:hsa00040	2	106
Jak-STAT signaling pathway	path:hsa04630	20	104
Fc epsilon RI signaling pathway	path:hsa04664	6	104
GnRH signaling pathway	path:hsa04912	1	100

Finally, we note the centrality of PPAR and adipocytokine signaling in the pathway network as the primary linkage between metabolism and cellular signaling pathways (Figure 5). As mentioned previously, these two pathways define the metabolic syndrome cluster in Figure 3. Several genetic and environmental factors are associated with each of these pathways, suggesting genetic and environmental modulators are critical to the role of these pathways in human disease progression, such as in metabolic syndrome phenotypes and cardiovascular disease.

## Conclusion

According to systems theory, although individual genes or environmental factors may be a critical component in the pathogenesis of a particular complex disease, it is ultimately the modulation of underlying pathways that the particular gene/environmental factor is a part of that determines the resultant phenotype. Here we have integrated gene centered knowledge from epidemiological and mechanistic environmental research in an attempt to discover the interplay between genetic and environmental mediators of phenotype at the pathway level. In addition, we have provided a higher order structure of pathway interconnectivity to build hypotheses of disease progression based on clusters of pathways defining phenotypes.

The methods and findings presented here open the door to a number of new hypotheses that can be explored regarding the genetic and environmental factors governing human disease. The results suggest retinol metabolism, Jak-STAT signaling, Toll-like receptor signaling, and adipocytokine signaling are key pathways that should be prioritized targets for high-throughput screening currently being implemented to improve toxicity testing [70,71]. For example, analysis of the metabolic syndrome subnetwork highlights the need for further epidemiological and mechanistic analyses of several compounds for their potential modulation of metabolic syndrome phenotypes, including plastic derivatives, synthetic and natural retinoids, pyrethrins and antipsychotic medications. In addition, the role of endocrine pathways in numerous phenotypes for which rates have increased over the last 30 years indicates a continued need to evaluate in greater detail the role of endocrine disruption in cancer, pregnancy and reproductive complications, and metabolic syndrome phenotypes. The multifactorial nature of complex diseases necessitates using knowledge-based, systems-driven evaluations, like the one presented here, for uncovering promising hypotheses for future research aimed at improving public health.

## Methods

### Characterization of Phenotype-Gene Relationships

The Genetic Association Database is an NIH supported gene-centered public repository of human association studies examining a wide range of human phenotypes, including non-mendelian common diseases, and is one of the largest databases of human disease associated polymorphisms currently available. All gene-phenotype relationships (N = 28,341) in the Genetic Association Database were downloaded (June 8, 2007). Phenotypes were further annotated to collapse synonyms, as well as group similar phenotypes into categories (see mapping used in Additional file 1). Only those phenotypes with at least 3 unique genes associated with it were analyzed further, resulting in 10,089 unique phenotype-gene relationships used in subsequent analyses.

### Characterization of Environment-Gene Relationships

The Comparative Toxicogenomics Database is an NIH supported public database that provides curated interactions between environmental factors and genes or proteins. Using either a MeSH concept or descriptor as the environmental factor identifier, all unique environmental factor-gene/protein relationships as of June 2007 (N = 47,025) were evaluated to define relationships between environmental exposures and human genes.

Annotation of MeSH concepts or descriptors was performed using the 2007–2008 MeSH browser  to identify any known biological actions of the environmental factors within the MeSH record. All environmental factors described in the Comparative Toxicogenomics Database fall within the Chemicals and Drugs [D] heading. To identify the most biologically relevant categorization, priority for annotation was set as follows: Noxae [D27.888.569], Physiological Effects of Drugs [D27.505.696], Therapeutic Uses [D27.505.954], Molecular Mechanisms of Action [D27.505.519]. If no information was available within these categories, then annotation by substance structure using all other trees under Chemicals and Drugs was implemented to annotate the given environmental factor.

### Evaluation of Gene-Pathway relationships

All sets of genes associated with a particular phenotype or environmental factor were analyzed for over-representation in specific molecular pathways found in the KEGG database [72] using Structurally Enhanced Pathway Enrichment Analysis (SEPEA), a novel pathway enrichment algorithm that incorporates relationships between nodes within a pathway using specific scoring rules described in detail elsewhere [13]. Briefly, the heavy ends scoring rule gives more importance to genes at the beginning (e.g. receptors) or end (e.g. transcription factors) of a pathway and the distance scoring rule gives more importance to those pathways where the perturbed genes (for a given condition) are close relative to each other in the pathway network. In this application, we use the SEPEA_NT3 method (see [13] for a detailed description). Broadly, the null hypothesis states that the distribution of the number of perturbed genes for a given condition in a specific pathway is not different from the distribution of a random set of genes chosen from all the genes involved in the KEGG pathways analyzed, in the context of the rules described above. Here we are incorporating a heavy ends scoring rule using a power function (0.5^δ^, with δ being the distance from a terminal node in the pathway), instead of a linear function as described in [13] Equation 8. Utilization of this power function emphasizes the underlying hypothesis of the biological importance of this rule in the final significance evaluation. We found that this emphasis resulted in a network with more clear separation of phenotypes and chemicals. In this analysis, those KEGG pathways developed based on a particular phenotype (disease pathways) were eliminated based on the potential redundancy of information found in the Genetic Association Database. Furthermore, only those KEGG pathways that had at least 3 human genes associated with them were analyzed.

### Pathway-Phenotype-Cluster

To determine relationships between human phenotypes based on polymorphisms associated with those phenotypes, phenotype p-values for KEGG pathway enrichment were clustered using Spearman Rank correlation with average linkage using Cluster version 2.11 and viewed using TreeView [73] downloaded July 2007 from 

### Phenotype-Environmental factor Network

Each phenotype-phenotype, phenotype-environmental factor, or environmental factor-environmental factor pair with at least two common significant pathways was assigned an edge. Network connectivity between phenotypes and environmental factors were determined for a range of SEPEA pathway enrichment p-value cut-offs and the sensitivity and specificity of the results as compared to manually curated, direct chemical-disease relationships found in the Comparative Toxicogenomics Database (CTD) database (downloaded in September 2008)[16]. This dataset contains direct chemical-disease relationships reported in the literature in human and animal model studies. We reduced this CTD database to those diseases found in GAD, which resulted in 1084 CTD relationships. We further collapsed these into the 8 broad disease categories. This analysis was used to establish the optimal pathway enrichment significance p-value cutoff of less than 0.00321 (which corresponds to a FDR ≤ 0.32 as computed using the Benjamini-Hochberg method [74]). This FDR is comparable to other pathway enrichment algorithm cut-offs (e.g. 0.25 for GSEA [75]), and is considered acceptable if one is primarily interested in hypothesis generation. The significance of these results was evaluated using the binomial cumulative distribution function where the probability (p_k_) of observing at least 226 (or 271) significant chemical-disease (or chemical-disease category) relationships by random chance was determined using the data for 4559 chemicals from CTD and for 204 diseases in GAD. Graphical representation of the network was determined using the edge weighted spring embedded algorithm in Cytoscape 2.5.0 downloaded Aug. 2007 [76] with the following parameters: spring strength = 5.0, spring rest length = 10.0, rest length of a disconnected spring = 1500, and strength of a disconnected spring = 0.06. Only those environmental factors with at least 2 genes associated with them were included in the final representation.

### Statistical Evaluation of Network

Each environmental factor or phenotype was labeled with one of 9 categories (Cardiovascular, Neurologic/Psychiatric, Neoplastic, Metabolic/Gastrointestinal, Immunologic, Hematologic, Endocrine/Reproductive, Pulmonary, Other) based on their classification in knowledge of the phenotype or Mesh categorizations for environmental factors as described above (See Additional file 2). The graph clustering coefficient method described in [17] was used to statistically evaluate the network generated. Briefly, p-values for the observed graph clustering coefficient for a given disease category (i) which has n(i) nodes associated with it in the network are based on choosing n(i) nodes randomly from the large network and computing the clustering coefficient for this random subset of nodes (based on 1000 permutations).

### Identification of enriched transcription factor binding sites in differentially expressed genes from microarray datasets

Up and downregulated gene lists from the microarray data as described in Table 1 was accessed from the publication associated with the datasets [30-36,77-84], or via downloading from GEO in Feb. or Nov. 2008. In the latter case, differentially expressed genes were identified using mattest and mafdr (Matlab 7.4.0.287 (R2007a)) with a fold change cutoff of 1.5 and a q value cutoff of 0.10. Lists of up or downregulated genes for each dataset were then submitted to DiRE , a transcription factor binding site (tfbs) enrichment optimization algorithm that identifies tfbs that are enriched in evolutionary conserved regions surrounding a given set of genes versus a background set of genes [45]. Based on analyses using tissue specificity gene expression datasets, the developers of DiRE show that an importance score cutoff of 0.10 is reasonable for achieving good specificity and precision [45], therefore we used this cutoff to identify those tfbs that are significantly enriched in sequence surrounding up or downregulated genes from each dataset (Additional File 4). Similar TRANSFAC binding sites [85] were then collapsed to avoid double counting tfs with similar binding sites (Additional File 6). Significance of consistently identifying a particular tf class across the metabolic or psychiatric datasets was tested using a binomial cumulative distribution, where the probability of observing at least *x *number of significant tfbs for a given tf across significance lists generated from the 12 (metabolic) or 9 (neuropsychiatric) datasets with a probability parameter equal to the mean frequency of occurrence of any tf that satisfied the importance score cutoff of 0.1 among all the datasets (0.137) was computed. To test the specificity of our findings as they relate to the metabolic syndrome datasets versus neuropsychiatric datasets analyzed, we compared the probability of finding a specific tfbs at least the observed number of times using the metabolic syndrome datasets versus the neuropsychiatric datasets and vice versa using a hypergeometric distribution.

### Generation of Pathway Interconnectivity Network

Connectivity between pathways was downloaded from KEGG (Aug. 16, 2007). Pathway network layout was generated using the force directed algorithm in Cytoscape 2.5.0 [76], where node size reflects the number of phenotypes associated with each pathway and node color gradation reflects the number of environmental factors associated with each pathway using a p-value cutoff of 0.003.

## Authors' contributions

JG and RT designed and implemented the research with important suggestions from CP. YZ and KB provided interpretation and management of GAD database and MR, AD, CM and CJM provided interpretation, data management, and annotation of CTD database. All authors have read and approved the final manuscript.

## Supplementary Material

Additional file 1 **Phenotype annotation of Genetic Association Database**. The spreadsheet provides original phenotype names from the Genetic Association Database mapped to the annotated phenotype names used in the present analysis. Click here for file

Additional file 2 **Annotation of nodes illustrated in Figure **3. The spreadsheet provides full chemical name annotation of the MeSH ID labels of nodes shown in Figure 3 as well as mapping to the 9 broad categories shown as colors in Figure 3. Click here for file

Additional file 3 **Annotation of metabolic syndrome and neuropsychiatric subnetworks**. The spreadsheet provides phenotypes and full chemical name annotation of the MeSH ID labels of nodes found within the 2 encircled subnetworks diagrammed in Figure 3. Click here for file

Additional file 4 **DiRE results for gene expression datasets described in Table **1. Full output from the DiRE program showing enriched tfbs for each of the microarray datasets represented in Figure 4. Click here for file

Additional file 5 **Comparison of results for Alzheimer's disease, Parkinson's disease and schizophrenia based on GAD or Genome Wide Association studies**. Full sets of genes found to be associated with Alzheimer's disease, Parkinson's disease, or schizophrenia based either on the GAD databse or a database only representing results from Genome Wide Association studies. Click here for file

Additional file 6 **Collapsed transcription factor binding site (tfbs) annotation based on similarity in matrices found in TRANSFAC**. A file containing original TRANSFAC matrix names collapsed to annotated matrix names used in the current analysis. Click here for file
